# Genetic structure and relationships of 16 Asian and European cattle populations using DigiTag2 assay

**DOI:** 10.1111/asj.12416

**Published:** 2015-08-11

**Authors:** Riku Yonesaka, Shinji Sasazaki, Hiroshi Yasue, Satoru Niwata, Yousuke Inayoshi, Fumio Mukai, Hideyuki Mannen

**Affiliations:** ^1^ Laboratory of Animal Breeding and Genetics, Graduate School of Agricultural ScienceKobe UniversityKobeJapan; ^2^ Tsukuba GeneTechnology LaboratoriesTsukubaJapan; ^3^ Kurabo Industries LtdNeyagawaJapan; ^4^ Yamaguchi Prefectural Agriculture & Forestry General Technology CenterYamaguchiJapan; ^5^ Wagyu Registry AssociationKyotoJapan

**Keywords:** Asia, cattle, DigiTag2 assay, genetic structure, SNP

## Abstract

In this study, we genotyped 117 autosomal single nucleotide polymorphisms using a DigiTag2 assay to assess the genetic diversity, structure and relationships of 16 Eurasian cattle populations, including nine cattle breeds and seven native cattle. Phylogenetic and principal component analyses showed that *Bos taurus* and *Bos indicus* populations were clearly distinguished, whereas Japanese Shorthorn and Japanese Polled clustered with European populations. Furthermore, STRUCTURE analysis demonstrated the distinct separation between *Bos taurus* and *Bos indicus* (*K*=2), and between European and Asian populations (*K*=3). In addition, Japanese Holstein exhibited an admixture pattern with Asian and European cattle (*K*=3‐5). Mongolian (*K*=13‐16) and Japanese Black (*K*=14‐16) populations exhibited admixture patterns with different ancestries. *Bos indicus* populations exhibited a uniform genetic structure at *K*=2‐11, thereby suggesting that there are close genetic relationships among *Bos indicus* populations. However, the Bhutan and Bangladesh populations formed a cluster distinct from the other *Bos indicus* populations at *K*=12‐16. In conclusion, our study could sufficiently explain the genetic construction of Asian cattle populations, including: (i) the close genetic relationships among *Bos indicus* populations; (ii) the genetic influences of European breeds on Japanese breeds; (iii) the genetic admixture in Japanese Holstein, Mongolian and Japanese Black cattle; and (iv) the genetic subpopulations in Southeast Asia.

## Introduction

In Asia, 258 cattle breeds have been reported, and some of these breeds (11%) are classified as being at the risk of extinction (Scherf 2000). In addition to these breeds, diverse native cattle, which are not classified as breeds, are raised in the fields of Asia. Therefore, understanding the genetic diversity, structure, relationships and evolutionary history of Asian cattle are important for improving future breeding and for the conservation of genetic resources.

The bovine whole genome sequence has been determined and over 2.2 million putative single nucleotide polymorphisms (SNPs) were identified (Bovine HapMap Consortium *et al*. 2009). Recently, high‐density SNP arrays, such as the Illumina Bovine SNP50 BeadChip (50 K) and the Illumina BovineHD BeadChip (770 K) (Illumina Inc., San Diego, CA, USA), were developed, providing powerful tools for genetic studies in cattle. However, using a smaller number of SNP markers is preferable to decrease the experimental costs in large‐scale investigations. DigiTag2 assay is a SNP typing system with a high conversion rate (>90%), high accuracy and low cost (Nishida *et al*. 2007), which has been successfully used as a typing method based on a moderate number of SNPs (Hijikata *et al*. 2012; Nishida *et al*. 2012; Shimogiri *et al*. 2012; Srilohasin *et al*. 2014).

In this study, we assessed the genetic diversity, structure and relationships of 470 unrelated cattle from 16 cattle populations using 117 autosomal SNPs for genotyping in a DigiTag2 assay. We investigated representative samples from native cattle in Southeast Asia and Northeast Asia, four Wagyu breeds, and three European cattle to elucidate the genetic structure and relationships of Asian cattle.

## Materials and Methods

### Animals

We collected 480 DNA samples from 16 Eurasian cattle breeds and populations of *Bos taurus* (Black Angus, Hereford, Japanese Holstein, Hanwoo, Tosa Japanese Brown, Higo Japanese Brown, Japanese Shorthorn, Japanese Polled, Japanese Black and Mongol native cattle) and *Bos indicus* (native cattle from Laos, Cambodia, Vietnam, Myanmar, Bhutan and Bangladesh). We collected samples of Black Angus and Hereford from Australia, Hanwoo from Korea and Japanese Holstein from nine prefectures in Japan: Tosa Japanese Brown from Kochi Prefecture, Higo Japanese Brown from Kumamoto Prefecture, Japanese Shorthorn from Iwate Prefecture, Japanese Polled from Yamaguchi Prefecture and Japanese Black from 17 prefectures in Japan. We selected 30 unrelated samples from each breed and population, and DNA was extracted from the blood, sperm or muscle.

### SNPs selection

The DigiTag2 assay is comprised of 96 SNPs in each SNP panel. In this study, two panels were developed and total of 192 SNPs were selected from the 2641 SNPs described by McKay *et al*. (2008) and Illumina BovineSNP50 BeadChip (Illumina Inc., San Diego, CA, USA). We employed an interval of >8.2 Mbp between two markers on the same autosome to avoid the linked loci (Khatkar *et al*. 2008). Among the 192 SNPs, 117 SNPs (52 SNPs on the first panel and 65 SNPs on the second panel) had a SNP call rate ≥95% and a minor allele frequency (MAF) ≥0.05, and were therefore utilized for analysis. Further information related to these SNP markers is provided in Table [Link]S1. In addition, 10 samples with individual call rates <0.05 (one Tosa Japanese Brown, three Higo Japanese Brown, one Japanese Shorthorn, one Japanese Black, two Myanmar cattle and two Bhutanese cattle) were excluded from the present study.

### Statistical analyses

The genotype and allele frequencies, the proportions of polymorphic markers and the expected (*He*) and observed (*Ho*) heterozygosities were calculated as indices of genetic diversity. Analyses of pairwise population differentiation (*Fst*) and within population differentiation (*Fis*) were performed with ARLEQUIN ver 3.5.1.2 (Excoffier *et al*. 2005).

To investigate the phylogenetic relationships among populations, the standard genetic distance of Nei (1972) was calculated from allele frequencies using PHYLIP 3.6 (Felsenstein 1989). Two types of phylogenetic trees were constructed from the distance matrix using the unweighted pair group method with arithmetic mean (UPGMA) (Sneath & Sokal 1973) and neighbor‐joining tree (NJ) algorithms (Saitou & Nei 1987) in MEGA 5.03 (Tamura *et al*. 2007). Principal component analysis (PCA) was performed with MVSP 3.1 (Kovach 2005) and the principal components were calculated from all the allele frequencies in each population.

The population structure and degree of admixture were inferred using STRUCTURE 2.3.4 (Pritchard *et al*. 2000), including prior information on populations. STRUCTURE uses Bayesian clustering of multi‐locus genotypes to assign individuals to populations, thereby estimating individual admixture proportions and inferring the number of parental populations (*K*) for a given sample. To obtain a representative value of *K* for modeling the data, we performed 16 independent runs of the Gibbs sampler for each K (1 ≤ *K* ≤ 17) with a 20 000 initial burn‐in used to minimize the effect of the starting configurations, followed by 100 000 Markov Chain Monte Carlo iterations, as recommended by Falush *et al*. (2007). We used the default settings, and the admixture model with correlated allele frequencies and the parameter of individual admixture alpha were set to be the same for all clusters.

## Results

We estimated the allele frequencies of 117 SNP markers in 16 populations. The mean MAF in each population ranged from 0.077 (Mongol) to 0.419 (Bangladesh) (Table 1). The mean MAF in *Bos taurus* populations (0.267) was higher than that in *Bos indicus* (0.195). We also calculated *Ho* and *He* within populations (Table 1). In the *Bos taurus* populations, *Ho* and *He* ranged from 0.315 (Higo Japanese Brown) to 0.394 (Mongol) and from 0.308 (Higo Japanese Brown) to 0.389 (Mongol), respectively. In contrast, those in *Bos indicus* populations were relatively low ranging from 0.229 (Laos) to 0.287 (Bhutan) for *Ho* and from 0.250 (Bangladesh) to 0.294 (Bhutan) for *He*. No deviations from the Hardy‐Weinberg equilibrium between *Ho* and *He* were observed in all populations (*P* > 0.05).

**Table 1:** Indices of genetic diversity within 16 cattle populations

Population	Samples no	MAF	*He*	*Ho*
*Bos taurus*				
ANG	30	0.291	0.377	0.383
HER	30	0.285	0.370	0.379
HOL	30	0.298	0.381	0.370
KOR	30	0.279	0.362	0.368
JBR (Tosa)	29	0.242	0.320	0.338
JBR (Higo)	27	0.230	0.308	0.315
JSH	29	0.253	0.336	0.352
JP	29	0.232	0.314	0.331
JB	30	0.260	0.343	0.326
MON	30	0.299	0.389	0.394
*Bos indicus*				
LAO	30	0.191	0.253	0.229
CAM	30	0.189	0.258	0.233
VIE	30	0.200	0.265	0.237
MYA	28	0.195	0.266	0.249
BHU	28	0.216	0.294	0.287
BAN	30	0.182	0.250	0.243
	470	0.240	0.318	0.315

ANG, Black Angus; HER, Hereford; HOL, Japanese Holstein; KOR, Hanwoo; JBR (Tosa), Tosa Japanese Brown; JBR (Higo), Higo Japanese Brown; JSH, Japanese Shorthorn; JP, Japanese Polled; JB, Japanese Black; MON, Mongolian native cattle; LAO, Laotian native cattle; CAM, Cambodian native cattle; VIE, Vietnamese native cattle; MYA, Myanmar native cattle; BHU, Bhutanese native cattle; BAN, Bangladeshi native cattle; MAF, mean minor allele frequencies; *He*, expected heterozygosity; *Ho*, observed heterozygosity.

We estimated Nei's genetic distance and *Fst* among all the cattle populations (Table 2). The genetic distances between *Bos taurus* and *Bos indicus* were high (0.140‐0.326), whereas those among Mongol cattle and *Bos indicus* populations were relatively low (0.140‐0.186). The highest value was observed between Japanese Polled and Bangladesh (0.326) and the lowest value was observed between Cambodia and Vietnam or Laos (0.008). In *Bos taurus*, the distance between Tosa Japanese Brown and Japanese Shorthorn was highest (0.203), and that between Mongol and Hanwoo was lowest (0.042). The distances among *Bos indicus* populations were relatively low (0.008‐0.041).

**Table 2:** Pairwise population differentiation (*Fst*) estimates (above diagonal) and Nei's genetic distance (below diagonal) among 16 cattle populations

Population†	ANG	HER	HOL	KOR	JBR (Tosa)	JBR (Higo)	JSH	JP	JB	MON	LAO	CAM	VIE	MYA	BHU	BAN
ANG	‐	0.160	0.147	0.168	0.205	0.205	0.158	0.147	0.194	0.123	0.320	0.312	0.307	0.302	0.263	0.326
HER	0.133	‐	0.105	0.167	0.194	0.209	0.194	0.242	0.211	0.130	0.319	0.320	0.315	0.310	0.279	0.331
HOL	0.126	0.086	‐	0.104	0.155	0.157	0.170	0.185	0.132	0.080	0.286	0.280	0.276	0.278	0.234	0.292
KOR	0.140	0.136	0.083	‐	0.141	0.119	0.209	0.184	0.074	0.049	0.300	0.290	0.282	0.276	0.237	0.303
JBR(Tosa)	0.162	0.147	0.117	0.100	‐	0.237	0.261	0.239	0.152	0.148	0.358	0.349	0.337	0.327	0.292	0.359
JBR(Higo)	0.158	0.158	0.115	0.081	0.166	‐	0.250	0.260	0.142	0.117	0.379	0.375	0.370	0.364	0.323	0.386
JSH	0.122	0.154	0.135	0.167	0.203	0.186	‐	0.257	0.234	0.167	0.354	0.343	0.345	0.340	0.298	0.357
JP	0.106	0.194	0.143	0.134	0.170	0.185	0.195	‐	0.189	0.172	0.393	0.387	0.381	0.388	0.336	0.406
JB	0.158	0.173	0.103	0.055	0.104	0.095	0.185	0.131	‐	0.078	0.319	0.312	0.304	0.291	0.250	0.319
MON	0.104	0.108	0.069	0.042	0.111	0.084	0.134	0.132	0.061	‐	0.246	0.243	0.236	0.224	0.188	0.257
LAO	0.255	0.249	0.216	0.223	0.267	0.283	0.272	0.308	0.234	0.176	‐	0.006	0.010	0.031	0.083	0.060
CAM	0.248	0.253	0.213	0.214	0.260	0.282	0.262	0.303	0.229	0.175	0.008	‐	0.005	0.022	0.056	0.043
VIE	0.246	0.251	0.210	0.208	0.248	0.280	0.268	0.299	0.222	0.170	0.009	0.008	‐	0.017	0.065	0.057
MYA	0.244	0.247	0.217	0.205	0.240	0.276	0.264	0.315	0.212	0.162	0.017	0.014	0.013	‐	0.041	0.038
BHU	0.213	0.228	0.183	0.178	0.217	0.244	0.231	0.264	0.183	0.140	0.041	0.030	0.034	0.024	‐	0.039
BAN	0.261	0.261	0.221	0.223	0.266	0.290	0.273	0.326	0.231	0.186	0.028	0.021	0.028	0.021	0.021	‐

† Population abbreviations are defined in Table 1.

**Figure 1:**
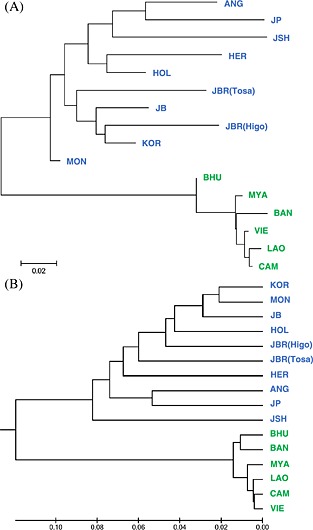
Phylogenetic constructions for 16 cattle populations. (A) neighbor‐joining tree, (B) unweighted pair group method with arithmetic mean tree. Population abbreviations are defined in Table 1. *Bos taurus* population indicated by blue and *Bos indicus* by green letters. Scale bar indicates the standard genetic distance of Nei (1972).

Two types of phylogenetic constructions, NJ tree (Fig. 1A) and UPGMA tree (Fig. 1B), among 16 cattle populations based on Nei's genetic distance illustrated the main divergence between the *Bos taurus* and *Bos indicus* clades. The *Bos indicus* populations were clustered close together. The Japanese Shorthorn and Japanese Polled were clustered with the European populations.

**Figure 2:**
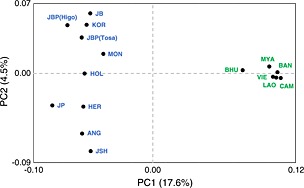
Principal component analysis (PCA) of 16 cattle populations. Population abbreviations are defined in Table 1. *Bos taurus* population indicated by blue and *Bos indicus* by green letters.

According to the PCA (Fig. 2), *Bos taurus* and *Bos indicus* were distinguished by PC1 (17.6%). European and Asian breeds were separated by PC2 (4.5%), whereas Japanese Polled and Japanese Shorthorn were clustered with European breeds. Japanese Holstein were located in an intermediate position between the European and Asian populations. *Bos indicus* populations were more closely clustered than *Bos taurus* populations as well as results of phylogenetic constructions.

**Figure 3:**
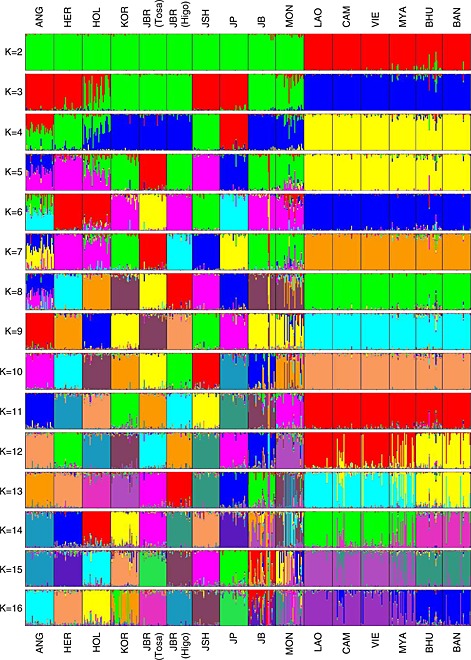
Genetic structure of 16 cattle populations based on 117 single nucleotide polymorphisms (SNPs) using STRUCTURE 2.3.4. Each individual is represented as a single vertical line and the proportion of the colored segment represents their estimated ancestry deriving from different populations. Population abbreviations are defined in Table 1.

Figure 3 shows the results of the STRUCTURE analysis with representative *K* values. We inferred the optimum number of genetic ancestral populations (*K*) by computing the log likelihood of the data (*LnP*(*D*)) and the second order rate of change of the likelihood function with respect to *K* (*ΔK*) (Evanno *et al*. 2005).

*Bos taurus* and *Bos indicus* were clearly distinguished at *K*=2 with the maximum *ΔK* value. At *K*=3, the separation between European and Asian *Bos taurus* populations was observed, although Japanese Shorthorn and Polled clustered with the European populations. At *K*=5, Tosa and Higo Japanese Brown were separated into two distinct groups. In addition, Japanese Holstein revealed admixture patterns with European and Asian populations at *K*=3‐5. At *K*=11, each *Bos taurus* population formed an independent cluster. At *K*=2‐11, the *Bos indicus* populations clustered into one group, but at *K*=12‐16, the Bhutan and Bangladesh cattle populations formed a separate cluster from the other *Bos indicus* populations. Mongolian, Japanese Black and Korean cattle showed admixture patterns that originated from different ancestries at *K*=13‐16, *K*=14‐16 and *K*=16, respectively.

## Discussion

In this study, we evaluated the genetic diversity, relationships and structure of 16 cattle populations using 117 autosomal SNPs. The genetic indices (Table 2), phylogenetic constructions (Fig. 1), PCA (Fig. 2) and STRUCTURE analysis (Fig. 3) determined the distinct subdivisions of *Bos taurus* and *Bos indicus* subspecies. These results agree with the historical background, which states that the cattle originated from independent domestication events in different locations in the Fertile Crescent (*Bos taurus*) and on the Indian subcontinent (*Bos indicus*).

Japanese Shorthorn and Japanese Polled clustered with European groups in the phylogenetic trees, PCA and STRUCTURE analyses (Figs 1, 2, 3). Japanese Shorthorn was established by crossing American Shorthorn and Nambu native cattle, which were distributed in the northeast region of Japan (Takeshima *et al*. 2002). Japanese Polled were established by crossing Japanese Black and Aberdeen Angus. Therefore, these Japanese breeds have stronger genetic influences from European breeds than the other Japanese breeds.

Although Tosa and Higo Japanese Browns were categorized as the same breed, they have different breeding improvement histories (Honda *et al*. 2006). The Japanese Browns were created mainly by crossing Korean native cattle with Japanese native cattle in the Meiji era. The Tosa Japanese Brown was then improved through the continuous introduction of Korean native cattle, whereas the Higo Japanese Brown was of Simmental and Devon breeds. This genetic background suggests that the genetic structure of Tosa and Higo Japanese Browns is similar to that of Korean cattle. At *K*=5 in STRUCTURE, Tosa Japanese Brown formed a distinct group from the Asian cattle group, including Korean cattle, Japanese Black and Mongolian cattle. At present, the Tosa Japanese Brown is maintained with a very small population size (<3000), which has likely resulted in genetic drift, thereby leading to a different genetic structure compared with the other Asian cattle populations.

Japanese Holstein had intermediate (Figs 1 and 2) and admixture (Fig. 3) topologies of European and Northeast Asian cattle. Japanese Holstein originated from Holstein‐Friesian cattle introduced from the United States and the Netherlands since the Meiji era. Tsuji *et al*. (2004) reported that Japanese Holstein have Asian unique mitochondrial types at a considerable frequency (18.3%), which have not been observed in European or North American Holsteins. This result supports that a number of Japanese Holstein cows are descend from native Japanese cattle, suggesting autosomal genetic admixture with Japanese native cattle as well as maternal mitochondrial DNA (mtDNA).

According to the STRUCTURE analysis at *K*=13‐16, Mongolian cattle exhibited admixture patterns that originated from different ancestries. Mongolian cattle strictly possess the morphological features of *Bos taurus* at present, but previous studies have detected the *Bos indicus* mitochondrial type in Mongolian cattle (Mannen *et al*. 2004; Lei *et al*. 2006; Jia *et al*. 2010; Yue *et al*. 2014). However, Mongolian cattle did not show any autosomal genetic admixture with *Bos indicus* (Fig. 3). Mongolia has a cool climate, which would not provide any selective advantage for heat‐tolerant *Bos indicus* cattle, so it is likely that the genetic influence of *Bos indicus* in Mongolian cattle has been reduced by natural and human selection. Mannen *et al*. (2004) reported high mtDNA diversity in Mongolian cattle, while Decker *et al.* (2014) also suggested the possibility of European introgression into East Asian cattle. Indeed, the continuity of the Asian and European Steppe facilitated the trade or plundering of various *Bos taurus* cattle during ancient times, and may have thereby generated the autosomal admixture due to different ancestries.

*Bos indicus* had close genetic relationships among the populations according to both phylogenetic trees (Fig. 1), the PCA (Fig. 2), and the uniformity of the genetic structure in the STRUCTURE analysis (Fig. 3), indicating lower genetic diversity in *Bos indicus* compared with *Bos taurus*. Bhutanese and Bangladeshi cattle populations formed a cluster distinct from the other *Bos indicus* populations at *K*=12‐16 in the STRUCTURE analysis (Fig. 3). The Arakan Mountains between Bangladesh and Myanmar divide the Indian subcontinent from Southeast Asia. These mountains prevented cattle migration and led to the differentiation of the genetic structures in these regions. The mitochondrial diversity described by Chen *et al*. (2010) also supports the different genetic structures of cattle in the Indian subcontinent and Southeast Asia.

In conclusion, we successfully determined the genetic diversity, structure and relationships of 16 cattle populations by using 117 autosomal SNPs genotyped in a DigiTag2 assay. Our results suggested: (i) the close genetic relationships among *Bos indicus* populations; (ii) the genetic influences of European breeds on Japanese breeds; (iii) the genetic admixtures in Japanese Holstein, Mongolian, Korean and Japanese Black cattle; and (iv) the presence of the genetic subpopulations in Southeast Asia. The genetic information obtained in this study would contribute to illuminating the genetic construction of Asian native cattle and facilitate future breeding improvements.

## Supporting information



Supporting info item
