# Supplementary material for: Genetic structure and relationships of 16 Asian and European cattle populations using DigiTag2 assay
Source: Anim Sci J. 2015 Aug 11;87(2):190–6. doi: 10.1111/asj.12416 (PMC5042107; doi:10.1111/asj.12416)
Supplement: Supplementary file 1 — Supporting info item [file ASJ-87-190-s001.docx]

**Table S1** 117 SNP information in this study

| SNP No. | | BTA | | Position (Mbp) |  | | | SNP No. | | BTA | | Position (Mbp) |  | | | SNP No. | | BTA | | Position (Mbp) |  |  |
| --- | --- | --- | --- | --- | --- | --- | --- | --- | --- | --- | --- | --- | --- | --- | --- | --- | --- | --- | --- | --- | --- | --- |
| SNP1-001 | | 1 | | 17.0 |  | | | SNP1-083 | | 25 | | 32.1 |  | | | SNP2-013 | | 2 | | 106.3 |  |  |
| SNP1-015 | | 4 | | 85.0 |  | | | SNP1-084 | | 25 | | 53.2 |  | | | SNP2-015 | | 2 | | 138.3 |  |  |
| SNP1-016 | | 4 | | 110.0 |  | | | SNP1-086 | | 26 | | 30.2 |  | | | SNP2-020 | | 3 | | 110.1 |  |  |
| SNP1-017 | | 5 | | 12.3 |  | | | SNP1-092 | | 28 | | 20.0 |  | | | SNP2-021 | | 3 | | 127.7 |  |  |
| SNP1-023 | | 6 | | 89.2 |  | | | SNP1-094 | | 29 | | 3.8 |  | | | SNP2-023 | | 4 | | 17.3 |  |  |
| SNP1-025 | | 7 | | 7.3 |  | | | SNP1-095 | | 29 | | 16.3 |  | | | SNP2-025 | | 4 | | 123.8 |  |  |
| SNP1-026 | | 7 | | 34.0 |  | | | SNP1-096 | | 29 | | 35.7 |  | | | SNP2-026 | | 5 | | 41.4 |  |  |
| SNP1-027 | | 7 | | 54.9 |  | | | SNP1-097 | | 1 | | 33 |  | | | SNP2-028 | | 5 | | 84.6 |  |  |
| SNP1-029 | | 8 | | 4.2 |  | | | SNP1-099 | | 1 | | 153.0 |  | | | SNP2-029 | | 5 | | 111.1 |  |  |
| SNP1-033 | | 9 | | 8.0 |  | | | SNP1-100 | | 2 | | 26.6 |  | | | SNP2-030 | | 5 | | 124.7 |  |  |
| SNP1-034 | | 9 | | 35.9 |  | | | SNP1-103 | | 3 | | 23.7 |  | | | SNP2-031 | | 6 | | 1.1 |  |  |
| SNP1-035 | | 9 | | 60.3 |  | | | SNP1-106 | | 4 | | 35.8 |  | | | SNP2-035 | | 6 | | 106.9 |  |  |
| SNP1-039 | | 10 | | 95.6 |  | | | SNP1-107 | | 4 | | 71.6 |  | | | SNP2-039 | | 7 | | 93.7 |  |  |
| SNP1-040 | | 11 | | 7.2 |  | | | SNP1-108 | | 4 | | 97.5 |  | | | SNP2-040 | | 8 | | 37.4 |  |  |
| SNP1-041 | | 11 | | 29.4 |  | | | SNP1-109 | | 5 | | 24.1 |  | | | SNP2-043 | | 8 | | 101.1 |  |  |
| SNP1-042 | | 11 | | 66.3 |  | | | SNP1-110 | | 5 | | 98.3 |  | | | SNP2-046 | | 9 | | 92.6 |  |  |
| SNP1-044 | | 12 | | 54.7 |  | | | SNP1-113 | | 6 | | 14.6 |  | | | SNP2-048 | | 10 | | 28.8 |  |  |
| SNP1-045 | | 12 | | 79.4 |  | | | SNP1-116 | | 7 | | 67 |  | | | SNP2-049 | | 10 | | 44.6 |  |  |
| SNP1-046 | | 13 | | 3.1 |  | | | SNP1-118 | | 8 | | 21.1 |  | | | SNP2-051 | | 10 | | 85.4 |  |  |
| SNP1-047 | | 13 | | 46.9 |  | | | SNP1-121 | | 9 | | 22.0 |  | | | SNP2-052 | | 10 | | 105.3 |  |  |
| SNP1-048 | | 13 | | 67.0 |  | | | SNP1-123 | | 9 | | 102.2 |  | | | SNP2-056 | | 11 | | 79.8 |  |  |
| SNP1-049 | | 14 | | 6.1 |  | | | SNP1-124 | | 10 | | 13.2 |  | | | SNP2-058 | | 11 | | 119.2 |  |  |
| SNP1-051 | | 14 | | 72.6 |  | | | SNP1-129 | | 11 | | 106.7 |  | | | SNP2-061 | | 12 | | 69.0 |  |  |
| SNP1-053 | | 15 | | 35.7 |  | | | SNP1-130 | | 12 | | 0.1 |  | | | SNP2-063 | | 13 | | 16.4 |  |  |
| SNP1-054 | | 15 | | 74.4 |  | | | SNP1-133 | | 13 | | 84.3 |  | | | SNP2-064 | | 13 | | 31.1 |  |  |
| SNP1-055 | | 16 | | 0.8 |  | | | SNP1-134 | | 14 | | 18.1 |  | | | SNP2-065 | | 13 | | 56.9 |  |  |
| SNP1-056 | | 16 | | 32.4 |  | | | SNP1-137 | | 15 | | 56.6 |  | | | SNP2-068 | | 14 | | 44.7 |  |  |
| SNP1-057 | | 16 | | 67.1 |  | | | SNP1-139 | | 16 | | 54.1 |  | | | SNP2-070 | | 15 | | 4.7 |  |  |
| SNP1-058 | | 17 | | 2.8 |  | | | SNP2-001 | | 1 | | 0.7 |  | | | SNP2-072 | | 15 | | 84.8 |  |  |
| SNP1-064 | | 19 | | 6.3 |  | | | SNP2-003 | | 1 | | 52.8 |  | | | SNP2-073 | | 16 | | 17.4 |  |  |
| SNP1-067 | | 20 | | 2.5 |  | | | SNP2-005 | | 1 | | 95.3 |  | | | SNP2-074 | | 16 | | 43.3 |  |  |
| SNP1-070 | | 21 | | 6.2 |  | | | SNP2-006 | | 1 | | 114.2 |  | | | SNP2-078 | | 18 | | 22.4 |  |  |
| SNP1-077 | | 23 | | 28.1 |  | | | SNP2-007 | | 1 | | 134.0 |  | | | SNP2-081 | | 19 | | 32.8 |  |  |
| SNP1-079 | | 24 | | 9.9 |  | | | SNP2-008 | | 2 | | 5.0 |  | | | SNP2-082 | | 19 | | 48.4 |  |  |
| SNP1-081 | | 24 | | 57.7 |  | | | SNP2-011 | | 2 | | 73.6 |  | | | SNP2-086 | | 20 | | 61.0 |  |  |
| SNP2-087 | | 20 | | 73.5 | | |  | SNP2-096 | | 22 | | 61.6 | | |  | SNP2-105 | | 26 | | 18.3 | | |
| SNP2-090 | | 21 | | 49.8 | | |  | SNP2-098 | | 23 | | 40.1 | | |  | SNP2-106 | | 26 | | 50.2 | | |
| SNP2-091 | | 21 | | 68.2 | | |  | SNP2-099 | | 23 | | 52.2 | | |  | SNP2-110 | | 28 | | 45.3 | | |
| SNP2-094 | | 22 | | 32.3 | | |  | SNP2-100 | | 24 | | 25.7 | | |  | SNP2-111 | | 29 | | 50.5 | | |
